# Exercise‐Induced Left Bundle Branch Block in a Patient With Syncope: A Case Report

**DOI:** 10.1002/ccr3.72500

**Published:** 2026-04-09

**Authors:** Akshay Manga, Ahmed Vachiat

**Affiliations:** ^1^ Department of Internal Medicine, Faculty of Health Sciences University of the Witwatersrand Johannesburg South Africa; ^2^ Division of Cardiology, Department of Internal Medicine, Faculty of Health Sciences University of the Witwatersrand Johannesburg South Africa

## Abstract

Exercise‐induced left bundle branch block (EI‐LBBB) is a rare phenomenon, particularly in patients without structural heart disease. Its pathophysiology remains poorly understood and there are no defined treatment protocols. We report a 77‐year‐old man with a history of syncope who developed EI‐LBBB during a cardiac stress test, with transient electrocardiographic changes that resolved at rest. Coronary angiography revealed only a mild–moderate (approximately 25%–50%) proximal left anterior descending artery stenosis, and the rest of his cardiac evaluation was unremarkable. The patient was commenced on an aerobic exercise program, and reported symptomatic improvement on follow‐up. Although EI‐LBBB is often linked to underlying structural heart disease and coronary artery disease, it can occur in patients with normal cardiac structure. This report summarizes an approach to evaluation and follow‐up of EI‐LBBB and reviews management strategies described in the literature, including exercise training in selected patients.

## Graphical abstract

Exercise‐induced left bundle branch block presenting with exertional syncope reveals a rate‐dependent conduction disease. Recognition should prompt targeted rhythm evaluation even in structurally normal hearts.

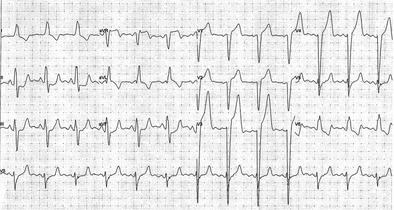

**Key Clinical Message**

Exercise‐induced left bundle branch block is rare and may occur in obstructive or non‐obstructive coronary disease, typically detected during exercise stress testing. Recognition of rate‐related LBBB should prompt evaluation for coronary and structural heart disease. In exertional syncope, consider ambulatory rhythm monitoring.

## 1 Introduction

Left bundle branch block (LBBB) is a conduction abnormality in which the electrical impulse in the left bundle branch is delayed, causing delayed activation of the left ventricle. LBBB is commonly associated with structural heart diseases such as coronary artery disease (CAD), valvular disorders and cardiomyopathies. However, exercise‐induced LBBB (EI‐LBBB) is uncommon and occurs in approximately 0.5% of patients undergoing an exercise stress test (EST) [1, 2]. This finding has been reported in patients with demonstrable cardiac abnormalities but is particularly rare in patients with no known structural heart disease [1].

This case report discusses a 77‐year‐old male with a history of syncopal attacks who developed EI‐LBBB during an EST. Cardiac evaluation revealed a mild–moderate (approximately 25%–50%) stenosis in the proximal left anterior descending (LAD) artery, while the rest of his cardiac evaluation remained normal. However, this degree of stenosis is not typically associated with ischemia, leading us to explore other possible explanations. The discussion is framed as an observational report and considers mechanisms and management approaches described in the literature.

## 2 Case History/Examination

A 77‐year‐old male with a medical history of hypertension, dyslipidemia, and recurrent episodes of exertional syncope was referred for cardiovascular evaluation. The patient experienced three episodes of exertional syncope over the last six months without preceding symptoms such as chest pain, palpitations, or prodromal dizziness. Each episode involved transient loss of consciousness lasting less than five minutes, with spontaneous recovery and no prolonged post‐event confusion. On physical examination, he was clinically stable and no signs of arrhythmias, heart failure, or respiratory distress were noted.

## 3 Methods/Investigations

**FIGURE 1:**
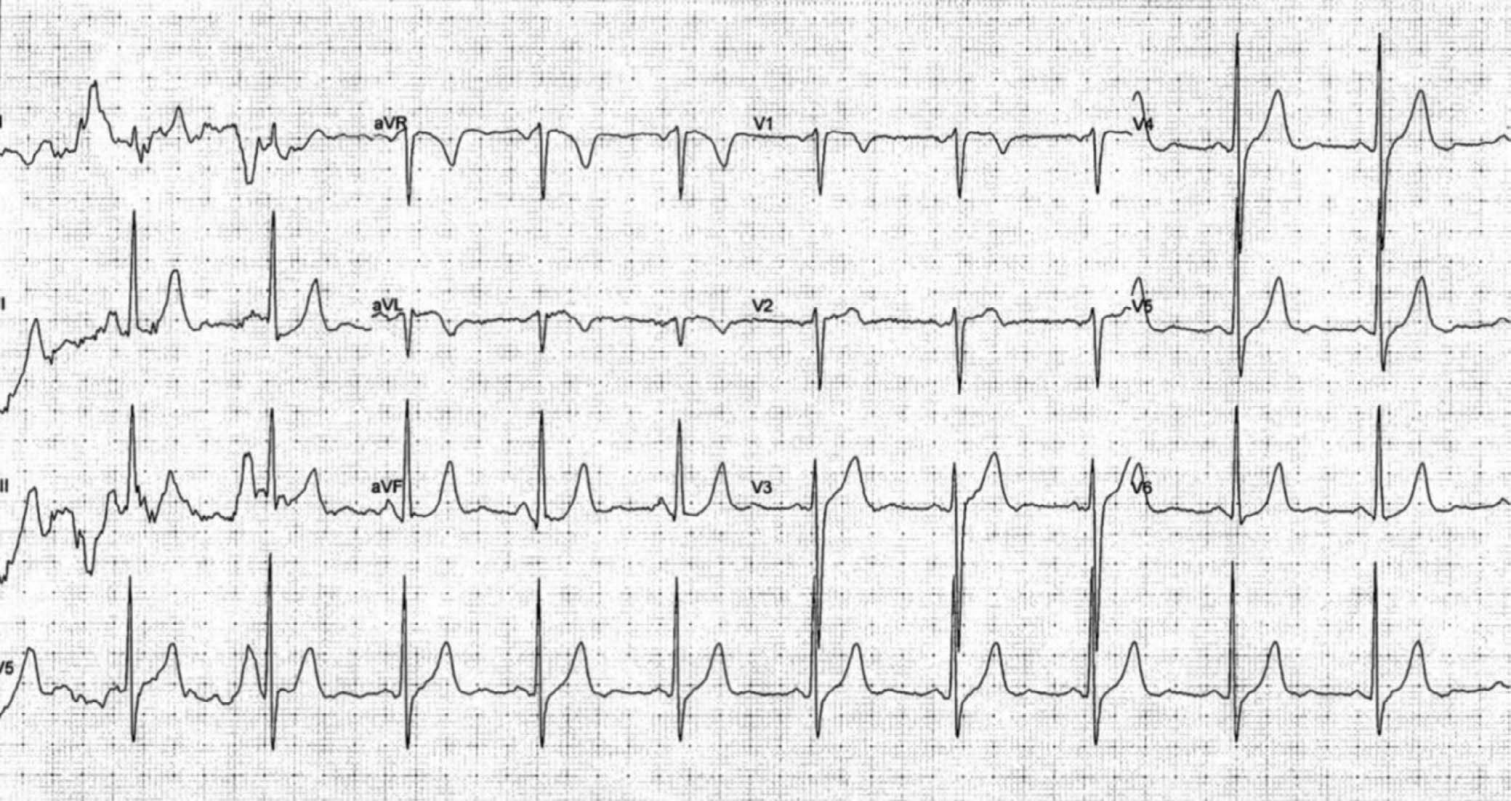
Resting electrocardiogram demonstrating normal baseline conduction.

**FIGURE 2:**
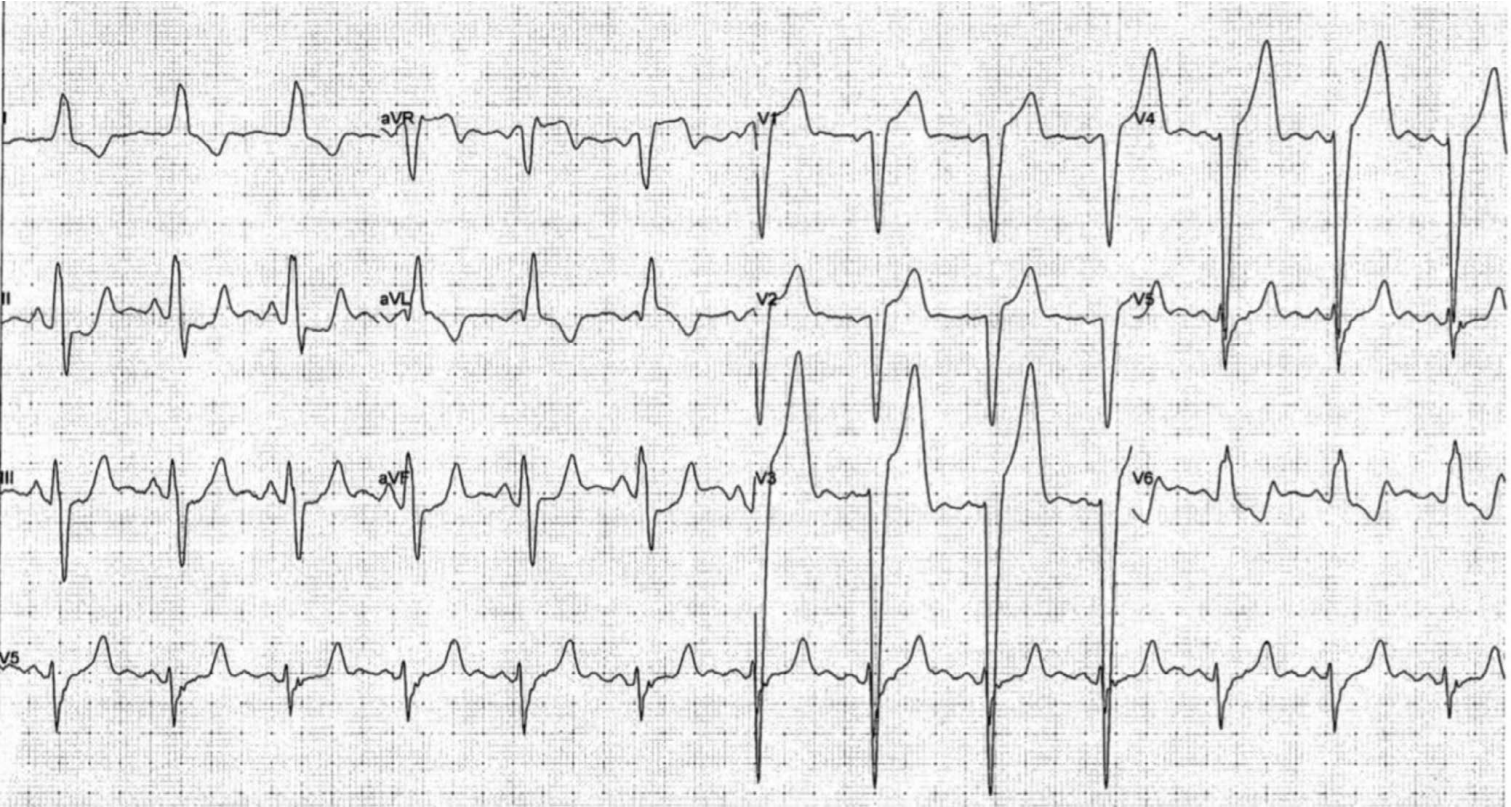
Electrocardiogram during exercise demonstrating new onset left bundle branch block.

His initial electrocardiogram (ECG) was unremarkable, with no arrhythmia or features of ischemia noted (Figure 1). The patient underwent an EST to further evaluate his cardiovascular fitness. The test was well tolerated initially, but at higher levels of exercise intensity at a heart rate just above 120 beats per minute, the ECG demonstrated a broad QRS complex with morphology consistent with complete LBBB (Figure 2), with no other ischemic findings such as angina or ST‐segment depression observed during EST. After cessation of exercise, the ECG normalized, and no other significant abnormalities were detected. Echocardiography was unremarkable, with a normal ejection fraction of 70%. A 24‐h ambulatory electrocardiogram (Holter) monitoring did not demonstrate significant arrhythmia, pauses, or high‐grade atrioventricular block; no symptomatic events were recorded during monitoring. The transient nature of the LBBB and the patient's history of syncope led to further investigation.

**FIGURE 3:**
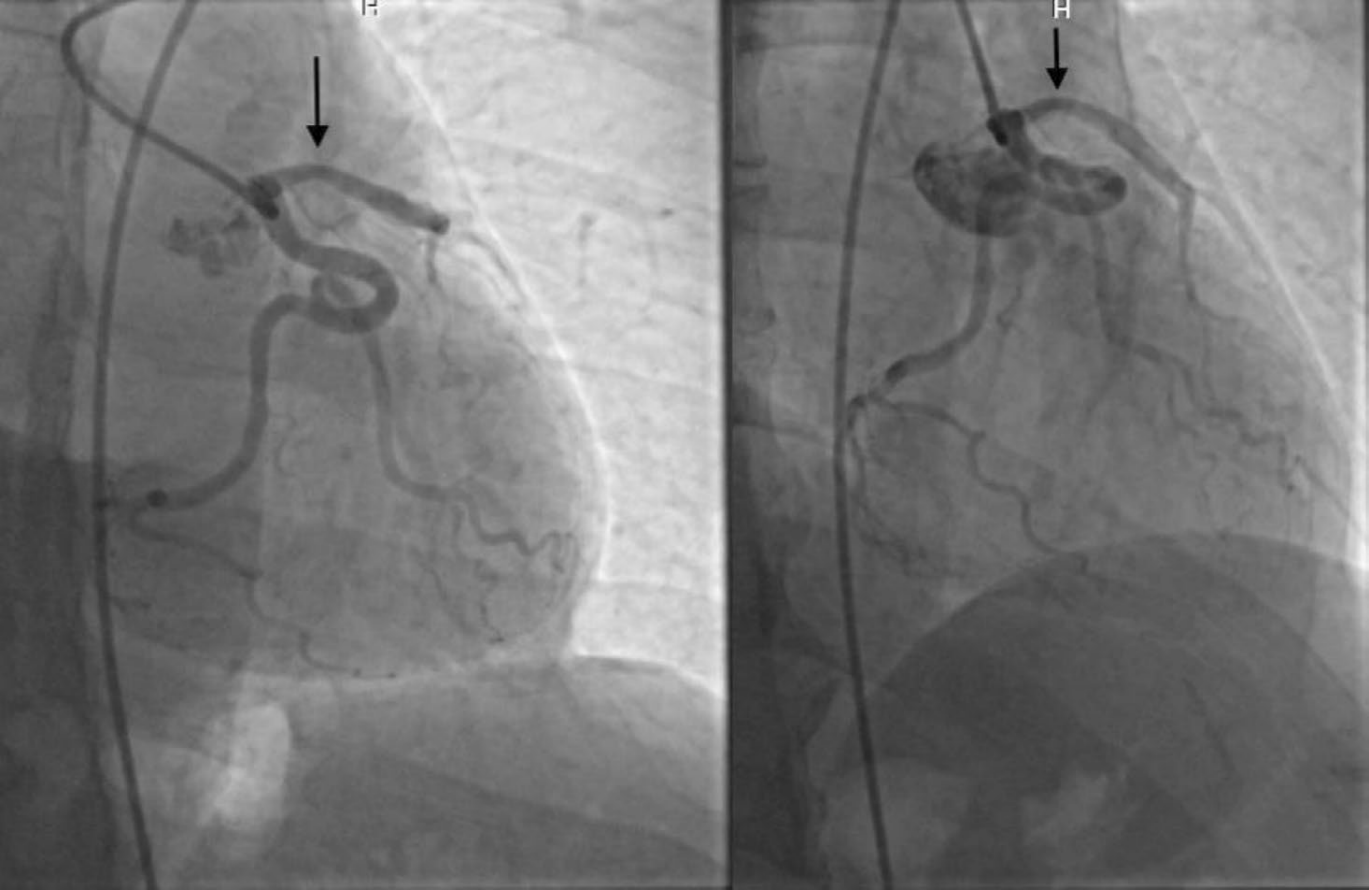
Coronary angiogram in right anterior oblique (RAO) caudal (left) and RAO cranial (right) projections. Both views demonstrate a mild–moderate proximal LAD stenosis (arrow).

Given the patient's risk profile, a diagnostic coronary angiogram was performed. By visual assessment, the proximal LAD demonstrated a mild–moderate (approximately 25%–50%) stenosis (Figure 3). As no physiological assessment of lesion significance was performed, its clinical significance in relation to EI‐LBBB remained uncertain.

## 4 Conclusion and Follow‐Up

The patient was reassured and advised to undertake a graduated aerobic exercise program, beginning with low‐intensity aerobic activity, and progressing gradually in intensity according to tolerance. On subsequent six‐month follow‐up, the patient was asymptomatic with no further presyncopal or syncopal symptoms.

## 5 Discussion

The mechanism of EI‐LBBB is poorly described. The pathophysiology appears to be multifactorial, involving dynamic changes in myocardial perfusion, conduction‐system recovery, and autonomic changes during exertion. Myocardial perfusion abnormalities have been observed even in patients without angiographically significant coronary artery disease (CAD). Candell‐Riera et al. demonstrated that individuals with EI‐LBBB and normal coronary arteries may still exhibit mild septal perfusion defects during exercise, despite the absence of obstructive disease on angiography [1]. This suggests that transient conduction delay may result from localized microcirculatory ischemia, a hypothesis supported by Loubeyre et al., who suggested that microvascular dysfunction could impair conduction during adrenergic stimulation [2]. Accordingly, the interplay of increased heart rate, sympathetic tone, and relative myocardial ischemia may result in rate‐dependent bundle‐branch delay. A causal relationship between EI‐LBBB and syncope could not be established in this case.

Alternatively, Vasey et al. proposed a predominant electrophysiological mechanism in which the primary cause is delayed recovery, with one bundle branch having a block in the third phase of the cardiac action potential, which can vary in length. Due to increased heart rate with exercise, stimuli from the proximal portion of the conduction pathway arrive before the fascicle has repolarized and block occurs [3]. This model explains both the reproducibility of the heart‐rate threshold that triggers EI‐LBBB and the improvement in conduction seen with exercise training.

Although LBBB is not typically associated with syncope, abrupt onset of EI‐LBBB may cause transient interventricular dysynchrony and a fall in cardiac output. In our patient, obstructive coronary artery disease, malignant arrhythmias and structural cardiomyopathy were excluded, however no single mechanism for syncope could be confirmed, highlighting the diagnostic uncertainty regarding exercise‐induced conduction abnormalities.

In patients with recurrent exertional syncope or persistent symptoms, further evaluation may be warranted. This may include prolonged rhythm monitoring, implantable loop recorder assessment, or electrophysiology study to exclude intermittent high‐grade conduction disease or other arrhythmic causes. In selected cases, device‐based therapy may also need to be considered.

While EI‐LBBB is a rare phenomenon, it does pose clinical concern. While the prognosis varies in the literature, it carries a twofold increase in all‐cause mortality [4, 5]. In most cases, it is associated with underlying structural heart disease, with a strong association with CAD; however, it has also been described in patients with no structural cardiac disease [1, 3, 4].

Even so, because of the lack of data on EI‐LBBB, the incidence of CAD in this population is uncertain and varies significantly in the literature [3, 4, 6]. It is, therefore, unclear whether the described risk associated with EI‐LBBB is due to underlying CAD. Stein et al. screened over 9000 individuals for EI‐LBBB. They demonstrated that although individuals with EI‐LBBB had a significantly higher all‐cause mortality rate compared to individuals without EI‐LBBB, it lost predictive ability when adjusted for the presence of CAD and/or heart failure [6]. This is consistent with other studies inferring that the prognosis of EI‐LBBB is favorable if there is no underlying structural heart disease [4, 6].

Candell Riera et al. echoed that view, illustrating that EI‐LBBB and CAD carried a worse prognosis. At the same time, those with normal coronary arteries had a favorable prognosis; however, in patients with EI‐LBBB without CAD, arrhythmias such as permanent LBBB and atrioventricular block developed on follow‐up in more than half of the patients [1]. This leads to the understanding that EI‐LBBB is not a benign finding, even in patients with normal coronary arteries.

Currently, there are no defined treatment protocols for EI‐LBBB, and management approaches are limited to case reports. Exercise training has proved effective in some patients, raising the heart rate threshold needed to induce the LBBB and subsequently improving symptoms [7, 8]. Pharmacological interventions such as low‐dose non‐selective beta‐blockers have been reported anecdotally, however its role remains uncertain [9].

More recently, permanent His bundle pacing (HBP) has emerged as a management strategy in symptomatic patients despite pharmacological treatment. It is predominantly employed in patients with “painful LBBB syndrome,” an uncommon variant of EI‐LBBB that results in angina [2, 10]. However, due to the lack of data, its role in patients without painful LBBB syndrome is unknown.

## 6 Conclusion

EI‐LBBB is a rare but important phenomenon, that has been associated in some cohorts with CAD and adverse outcomes. Still, it is also observed in patients with normal coronary arteries. While those with normal coronary arteries tend to have a more favorable prognosis, some have reported the development of permanent arrhythmias requiring intervention, suggesting adequate surveillance of these patients. Further investigation is needed to define optimal treatment protocols for EI‐LBBB. However, exercise training, pharmacological treatments and HBP could be explored in symptomatic patients.

## Author Contributions

**Akshay Manga:** conceptualization, data curation, formal analysis, investigation, methodology, project administration, writing – original draft. **Ahmed Vachiat:** conceptualization, formal analysis, methodology, supervision, writing – review and editing.

## Funding

The authors have nothing to report.

## Ethics Statement

As a single‐case report with the patient's signed consent, no other ethical review was required.

## Consent

Written informed consent was obtained from the patient for the publication of this case report.

## Conflicts of Interest

The authors declare no conflicts of interest.

## Data Availability

The data supporting the findings of this study are available from the corresponding author upon reasonable request. Due to ethical and privacy considerations, patient‐related data and genetic information have been anonymized and are not publicly accessible.
